# CHD1L is associated with poor survival and promotes the proliferation and metastasis of intrahepatic cholangiocarcinoma

**DOI:** 10.3892/or.2019.7262

**Published:** 2019-08-05

**Authors:** Shimiao Li, Yi Chai, Yanbao Ding, Tinghao Yuan, Changwen Wu, Changwen Huang

Oncol Rep 42: 657-669, 2019; DOI: 10.3892/or.2019.7174

Subsequent to the publication of the above paper, the authors have realized that the second affiliation for the second named author, Yi Chai, was not included with the affiliations. His second affiliation should have been listed as: “Department of Neurosurgery, Yuquan Hospital, School of Clinical Medicine, Tsinghua University, Beijing 100040, China.” Therefore, the author affiliations for this paper should have appeared as follows:

SHIMIAO LI^1*^, YI CHAI^2,3*^, YANBAO DING^4^, TINGHAO YUAN^4^, CHANGWEN WU^5^ and CHANGWEN HUANG^1^

^1^Department of Hepatobiliary Surgery, Jiangxi Provincial People's Hospital, Nanchang, Jiangxi 330006; ^2^Department of Neurosurgery, Yuquan Hospital, School of Clinical Medicine, Tsinghua University, Beijing 100040; ^3^Department of Neurosurgery, Shangrao People's Hospital, Shangrao, Jiangxi 334000; ^4^Department of Hepatobiliary Surgery; ^5^Department of Urology Surgery, The Second Affiliated Hospital of Nanchang University, Nanchang, Jiangxi 330006, P.R. China

The authors regret that this was not corrected prior to the publication of the paper, and apologize to the readers for any inconvenience caused.

